# The Integrity of the Cytokinesis Machinery under Stress Conditions Requires the Glucan Synthase Bgs1p and Its Regulator Cfh3p

**DOI:** 10.1371/journal.pone.0042726

**Published:** 2012-08-15

**Authors:** Mohammad Reza Sharifmoghadam, M.-Ángeles Curto, Marta Hoya, Nagore de León, Rebeca Martin-Garcia, Cristina Doncel, M.-Henar Valdivieso

**Affiliations:** 1 Departamento de Microbiología y Genética/Instituto de Biología Funcional y Genómica, Universidad de Salamanca/Consejo Superior de Investigaciones Científicas, Salamanca, Spain; 2 Faculty of Veterinary Medicine, University of Zabol, Zabol, Sistan and Baluchestan, Iran; Cancer Research UK London Research Institute, United Kingdom

## Abstract

In yeast, cytokinesis requires coordination between nuclear division, acto-myosin ring contraction, and septum synthesis. We studied the role of the *Schizosaccharomyces pombe* Bgs1p and Cfh3p proteins during cytokinesis under stress conditions. Cfh3p formed a ring in the septal area that contracted during mitosis; Cfh3p colocalized and co-immunoprecipitated with Cdc15p, showing that Cfh3p interacted with the contractile acto-myosin ring. In a wild-type strain, a significant number of contractile rings collapsed under stress conditions and this number increased dramatically in the *cfh3Δ, bgs1cps1-191*, and *cfh3Δ bgs1/cps1-191*. Our results show that after osmotic shock Cfh3p is essential for the stability of the (1,3) glucan synthase Bgs1p in the septal area, but not at the cell poles. Finally, cells adapted to stress; they repaired their contractile rings and re-localized Bgs1p to the cell surface some time after osmotic shock. A detailed analysis of the cytokinesis machinery in the presence of KCl revealed that the actomyosin ring collapsed before Bgs1p was internalized, and that it was repaired before Bgs1p re-localized to the cell surface. In the *cfh3Δ, bgs1/cps1-191*, and *cfh3Δ bgs1/cps1-191* mutants, which have reduced glucan synthesis, the damage produced to the ring had stronger consequences, suggesting that an intact primary septum contributes to ring stability. The results show that the contractile actomyosin ring is very sensitive to stress, and that cells have efficient mechanisms to remedy the damage produced in this structure.

## Introduction

Cytokinesis is the final stage of cell division and results in a roughly equal distribution of organelles in each of the two daughter cells. In the fission yeast *Schizosaccharomyces pombe*, cytokinesis requires the positioning of the division plane, the assembly and contraction of an actomyosin ring, the synthesis and the degradation of a division septum, and the coordination of all these processes with nuclear division [1]–[11].

The division plane coincides with the position of the nucleus in order to ensure that both daughter cells will receive an equal number of chromosomes. Microtubules emplace the nucleus at the medial region of the cell, and Mid1p and the kinase Plo1p promote the recruitment and assembly of the contractile actomyosin ring (CAR) at the cell cortex around the nucleus [11]–[16]. First, the type-II myosin heavy-chain Myo2p, its light chains Rlc1p and Cdc4p, and Rng2p arrive at the equator of the cell in a Mid1p-dependent manner. Then, the PCH protein Cdc15p and the formin Cdc12p become incorporated to the ring and promote the recruitment of certain actin-interacting proteins that initiate the polymerization and compaction of actin into a ring. Maturation of the ring is accompanied by the incorporation of additional proteins to this structure [4], [17]–[23].

Once the CAR has been assembled, its contraction is initiated by the activity of a cascade of protein kinases (the SIN pathway, from Septation Initiation Network) that assembles at the spindle pole body. Mutants in components of this pathway are able to assemble a CAR but this ring is unstable and does not contract [5], [10], [22]. In the case of yeasts, ring contraction is accompanied not only by the incorporation of new plasma membrane but also by the synthesis of a septum composed of cell wall material [8], [24]. In fission yeast, the primary septum, composed of linear and branched (1,3) glucan, is surrounded by a secondary septum that has a composition similar to that of the lateral cell wall [25], [26]. Bgs1p plays a relevant role in cytokinesis because it is the (1,3)glucan synthase responsible for the synthesis of linear (1,3)glucan and for the integrity of the primary septum [27]. Finally, the septum needs to be degraded in order to allow both daughter cells to separate. (1,3)- and -glucanases have been implicated in cell separation [28]–[30]. Septins and the exocyst are required for the correct localization of glucanases [31].

Cfh3p is similar to *S. cerevisiae* Chs4p, a scaffold protein that attaches the chitin synthase Chs3p to the septin ring. Cfh3p is a protein that regulates the activity of Bgs1p by stabilizing it at the cell surface [32]. Cfh3 and Chs4 proteins share the presence of tandem SEL1 domains, a subfamily of TPR domains that are present in proteins involved in multiprotein complexes required for signal transduction [33]. Here we show that stress collapses the cytokinesis machinery and that Bgs1p and its regulator Cfh3p are required to ensure the stability of the cytokinesis apparatus under these conditions. The results point to the notion that Cfh3p acts as a scaffold that ensures the stability of Bgs1p at the septal area, so that linear (1,3)glucan can be synthesized even under unfavorable conditions.

## Results

### Overexpression of *cfh3^+^* produces an abnormal distribution of proteins involved in cytokinesis

Previous results had shown that *cfh3^+^* overexpression results in a defect in cytokinesis [34]. In order to gain further information about the role of Cfh3p in this process, we analyzed the distribution of proteins involved in the different steps of cytokinesis in cells overexpressing *cfh3^+^*. We focused our attention on the distribution of CAR components (actin, the myosin light chains Cdc4p and Rlc1p, and Cdc15p); on a protein that links the CAR to the plasma membrane (Chs2p), and on some proteins involved in cell separation (the septin Spn3p and the glucanases Agn1p and Eng1p). The results showed that an excess of Cfh3p produced alterations in the localization of all these proteins (figure S1). Since Cfh3p regulates the activity of Bgs1p [32], we wondered whether this alteration of cytokinesis was due to a hyperactivation of Bgs1p. In fact, in cells overexpressing *cfh3^+^* Bgs1p was not only observed at the cell surface of cell poles and septal area, as in the WT strain, but across the whole of the cell periphery (figure S2, A). However, the following results argue against the hypothesis that an altered Bgs1p regulation would be the cause of the defects in cytokinesis exhibited by cells overexpressing *cfh3^+^*. 1) -glucan synthase activity did not increase in these cells (not shown), and 2) overexpression of *bgs1^+^* from the 3X*nmt1^+^* promoter produced cells with an abnormal morphology that sometimes lysed; however, these cells were not chained, branched or multiseptated (figure S2, B). These results suggested that the interference of Cfh3p with cytokinesis was not a consequence of a hyperactivation of Bgs1p. The specificity of the interaction of *cfh3^+^* overexpression with the contractile ring was supported by the fact that the multiseptation phenotype was not observed in *cdc15–140* and SIN mutants, which cannot assemble stable CARs, whereas it was observed in septin mutants (figure S3), in which CARs assemble and contract and septa are synthesized but not dissolved owing to glucanase misregulation [31]. It has been suggested that the function of *cfh3^+^* would be to regulate Chs2p [34], a protein similar to chitin synthases that lacks such catalytic activity [35] and whose overexpression leads to cytokinesis defects [36]. As shown in supplemental figure S3, the phenotype of *cfh3^+^* overexpression was produced in cell lacking *chs2^+^*, and vice versa. Taken together, these results suggested that Cfh3p might interact physically with the CAR, such that a high concentration of this protein would disturb the structural/mechanical properties of the ring.

### Cfh3p accumulates at the cell poles and septal area

**Figure 1:**
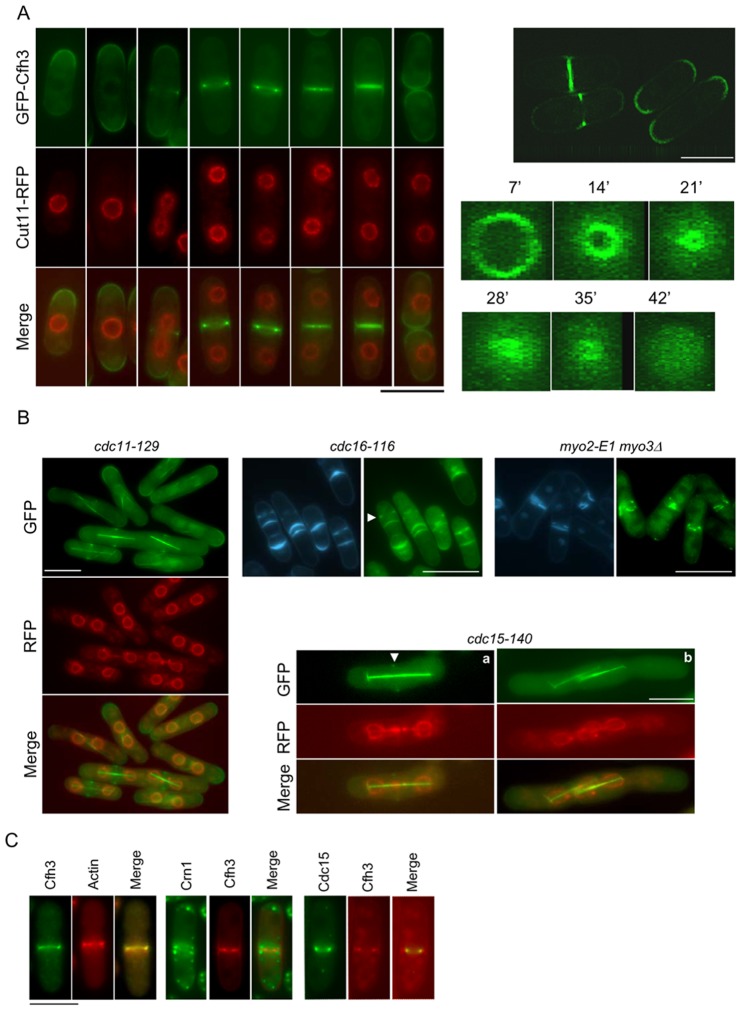
Cfh3p accumulates at the cell poles and septal area. A. Localization of Cfh3p in a WT strain. Left panel, micrographs of different cells from a strain bearing GFP-Cfh3 and Cut11-RFP; the pictures were taken with a conventional fluorescence microscope. Right panels, micrographs of a cell bearing GFP-Cfh3 taken with a confocal microscope; the upper panel shows a z-section while the lower panels show three-dimensional reconstructions of stacks of z-series taken along time to show CAR contraction; the numbers indicate the time-points (in minutes) at which the cell was photographed. B. Localization of Cfh3p in different strains. In the case of the *cdc15–140* and *cdc11–129* mutants, the cells expressed RFP-tagged Cut11p and GFP-tagged Cfh3 and Atb2 proteins; the cells were photographed after 3 hours of incubation at 36°C; the arrowhead in the *cdc15–140* panel points to a weak GFP-Cfh3 signal (a, and b depict different cells from the same culture). In the case of the *cdc16–116* and *myo2-E1 myo3Δ* strains, the left panels correspond to staining with Hoechst 33258 and the right panels to the GFP fluorescence. The arrowhead in the *cdc16–116* panels points to a Cfh3p ring that corresponds to a growing septum. C. Cfh3p co-localizes with actin and with Cdc15 at the CAR. Left panels, GFP-Cfh3p and rhodamine-phalloidin images. Central panels, GFP-tagged coronin (Crn1) and RFP-tagged Cfh3p images. Right panels, GFP-tagged Cdc15p and RFP-tagged Cfh3 images. Bar, 10 µm.

According to the databases, Cfh3p is a prenylated protein. Accordingly, it was expected to localize to the cell surface. A GFP-Cfh3 fused protein was observed at the cell poles and at the midzone of the cell in the WT strain, (figure 1 A), in agreement with the localization described by Matsuo *et al.* using immunofluorescence analyses [34]. A strain bearing GFP-Cfh3 and Cut11-RFP showed that the Cfh3p signal accumulated at the cell poles of interphase cells. In mitotic cells, Cfh3p was observed at the cell equator before the nuclei separated; at later times, a strong Cfh3p signal accumulated at the cell equator; this signal contracted along time, and remained at the septal area before the cells separated. After cell separation, Cfh3p was observed at the cell poles (figure 1 A, left panels). Confocal microscopy confirmed that Cfh3p accumulated at the cell poles and septal area (figure 1 A, right panels). A time-lapse experiment using confocal microscopy allowed us to perform a more detailed analysis of Cfh3p localization to the cell midzone; we observed that Cfh3p was initially assembled as a ring and that this ring contracted during cytokinesis, leaving behind a fluorescent signal that formed a plaque when the leading ring was disassembled at the end of contraction (figure 1 A, lower right panel). This result suggested that Cfh3p was associated with both the contractile ring and the plasma membrane.

We analyzed Cfh3p localization at the cell midzone in mutants affected in different stages of cytokinesis: CAR assembly and contraction (*cdc4–8*, *rlc1Δ*, *myo2-E1 myo3Δ*, *cdc15–14*0, and *chs2Δ*), SIN signaling (*cdc11–119* and *cdc16–116*), septum synthesis (*cps1-191*) and cell separation (*spn3Δ* and *spn4Δ*). When the SIN-defective *cdc11–119* mutant was incubated for 3 hours at 36°C, the strongest Cfh3p signal was detected at the cell poles of interphase cells, and at the cell midzone of mitotic cells (figure 1 B). In a *cdc16–116* mutant, in which the SIN signal does not turn off, Cfh3p localized to the edge of the growing septa and it remained at the septal area after the septa had been completed. Thus, Cfh3p can arrive at the cell midzone in the absence of the SIN pathway but it requires that the SIN signal must be turned off for it to be removed from the cell equator after mitosis. Cfh3p localized to the cell equator of the cells in the myosin *myo2-E1 myo3Δ*, *cdc4–8*, and *rlc1Δ* mutants, although the signal was not uniform, in accordance with the altered CARs in these mutants (figure 1 B and results not shown). In a *cdc15–140* mutant, a weak GFP-Cfh3 signal was observed at the inter-nuclei area of about 30% of mitotic cells (arrowhead in panel *a* of figure 1 B); this result suggested that GFP-Cfh3p was able to arrive at the cell midzone in these cells but was not able to remain there for a long time. In the *cps1-191*, *chs2Δ, spn3Δ*, and *spn4Δ* mutants Cfh3p localized to the cell equator. In sum, Cfh3p localization to the cell equator was independent of the myosin components of the CAR, the glucan synthase Bgs1p, and the septins; Cdc15p was required to stabilize Cfh3p at the cell equator and the SIN activity regulated the removal of Cfh3p from the midzone after mitosis. Time-lapse experiments using cells that expressed GFP- and RFP-tagged Cdc15p, Cfh3p, and Bgs1p indicated that Cfh3p arrived at the cell midzone after Cdc15p and before Bgs1p (figure S4). These results were in agreement with a role of Cfh3p in CAR maturation/contraction and/or in septum synthesis.

We also analyzed the localization of proteins involved in cytokinesis in a *cfh3Δ* mutant; we found that Cdc15-GFP, Chs2-GFP, GFP-Bgs1p, Spn3-GFP, and Eng1-GFP localized correctly in the absence of Cfh3p (not shown). However, we observed that a number of the Cdc15-GFP and the GFP-Cdc4 rings were asymmetric or broken.

Finally, we performed co-localization analyses between Cfh3p and CAR proteins. It was found that GFP-Cfh3 and actin (stained with rhodamine-phalloidin) co-localized to the contractile ring (figure 1 C, left panels). Observation of a strain bearing GFP-fused coronin, a protein that associates with actin patches [37], and RFP-Cfh3p revealed that Cfh3p did not co-localize with actin patches (figure 1 C, central panels), indicating that Cfh3p is not associated with all actin-containing structures. RFP-Cfh3p co-localized with the CAR-associated protein Cdc15p fused to GFP (figure 1 C, right panels).

### *cfh3Δ* mutants show a genetic interaction with mutants defective in CAR assembly and contraction

**Figure 2:**
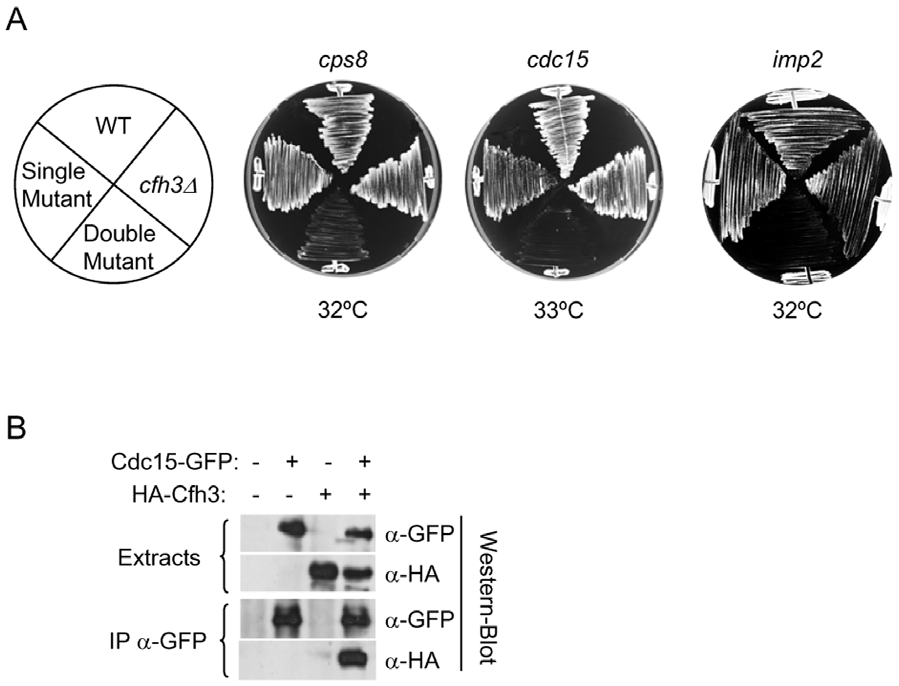
Cfh3p is a ring-associated protein. A. The *cfh3Δ* mutant shows a genetic interaction with mutants affected in CAR assembly/contraction. Cells from the indicated strains were streaked onto YES plates and incubated at the indicated temperatures for 2 days. B. Cfh3p and Cdc15p co-immunoprecipitate. Cell extracts from strains carrying Cdc15-GFP and/or HA-Cfh3 fusion proteins were analyzed by Western blotting using monoclonal anti-GFP (-GFP) or anti-HA (-HA) antibodies before (Extracts) or after immunoprecipitation (IP) with a polyclonal anti-GFP antibody.

In a previous work, we determined that *cfh3Δ* was synthetic sick with the *cdc14–114* SIN-defective mutant and with the *cps1-191* mutant [32], which involved Cfh3p in septum synthesis. The *cfh3Δ* mutants did not show a genetic interaction with mutants affected in the myosin component of the CAR or with septin mutants. Here, we extended the analysis of genetic interactions to analyze the functional relationship between *cfh3Δ* and mutants affected in other CAR components. Thus, we constructed double mutants between *cfh3Δ* and *cps8–188* (a strain carrying a point-mutation in the *act1^+^* gene, coding for actin), and between *cfh3Δ* and *cdc15–140* and *imp2Δ* strains (carrying mutations in PCH-family proteins required for CAR function). The WT strain and the mutants carrying single or double mutations were streaked onto YES plates and incubated at different temperatures (22°C to 37°C). It was found that the *cfh3Δ cps8–188*, *cfh3Δ cdc15–140*, and *cfh3Δ imp2Δ* strains were more thermosensitive than the corresponding single mutants (figure 2 A). Thus, *cfh3Δ* showed a genetic interaction with some mutants affected in CAR assembly and/or contraction, pointing to a role of Cfh3p in these processes.

### Cfh3p is a CAR-associated protein

The facts that *cfh3^+^* overexpression produced an abnormal distribution of proteins involved in different steps of cytokinesis, and that Cfh3p localized to the septal area as a contractile ring, suggested that Cfh3p might associate with the CAR. This possibility was analyzed by performing a co-immunoprecipitation experiment. We incubated cell extracts from strains carrying HA-Cfh3, Cdc15-GFP, or both tagged proteins, in the presence of polyclonal anti-GFP antibody. Following this, we performed Western blotting analyses using monoclonal anti-GFP or anti-HA antibodies. In parallel, total cell extracts from the same strains were analyzed by Western blotting to detect the input of Cfh3p or Cdc15p. As shown in figure 2 B, HA-Cfh3 was detected in anti-GFP immunoprecipitates from the strain bearing both tagged proteins, but not from the control strains, pointing to a physical interaction between Cfh3p and a CAR component or a CAR-associated protein.

### Contractile rings in the *cfh3Δ* mutant are sensitive to stress

Since the above results suggested that Cfh3p might play some role in the assembly and/or contraction of the CAR, we carried out time-lapse experiments using strains bearing both the Cdc15 ring protein and the Hht2 histone fused to GFP in order to visualize the progression of nuclear division. In this way, the photographs could be compared at the same time points. Our initial results showed that the time for CAR assembly and contraction for the control strain was about 40±3 minutes (n = 10), in agreement with previous results [36], while for the *cfh3Δ* strain it was about 75±9 minutes (n = 10). This result suggested that Cfh3p might play a relevant role in CAR assembly/contraction; however, since the *cfh3Δ* mutant did not show either a delay in the generation time or an increase in the number of septated cells, we wondered whether this surprising result might be a consequence of the method used to prepare the samples, which involved centrifugation of the cells and their mixing with melted solid medium kept at 42°C. Indeed, when the samples were prepared by filtering the cells and spreading them onto solid YES medium layered on the slides, the time for ring assembly and contraction in both strains was about 40±3 minutes (n = 10).

**Figure 3:**
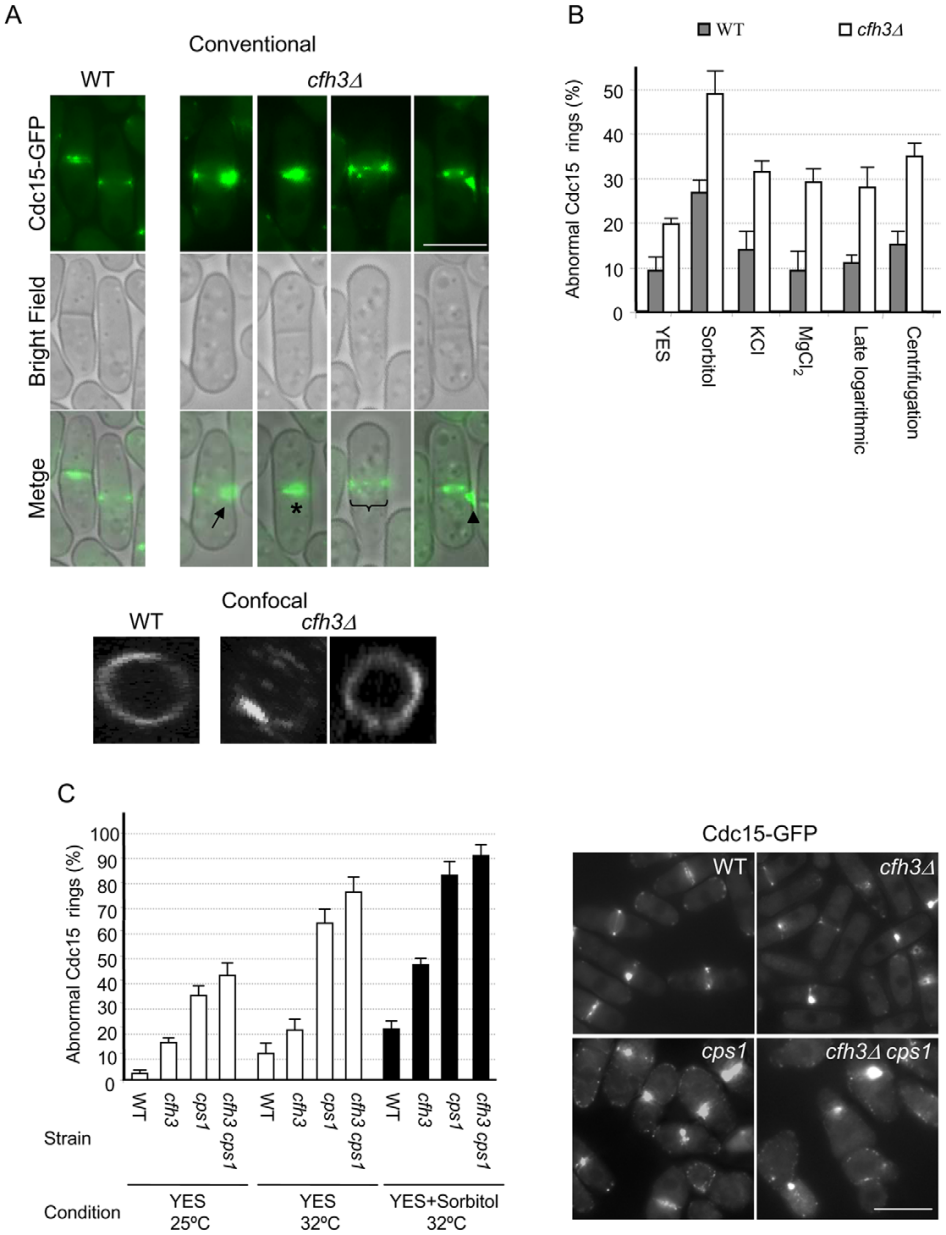
Cfh3p and Bgs1p are required for CAR integrity under stress conditions. A. Conventional and confocal fluorescence microscopy of WT or *cfh3Δ* cells treated with 1 M KCl for 15 minutes and collected by centrifugation. In the panels showing the bright field and fluorescence overlaid images, the arrow points to an asymmetric ring; the asterisk shows a ring that did not disassemble after the septum had been completely synthesized; the bracket marks a broken ring, and the arrowhead points to an abnormal accumulation of the Cdc15 protein at the cell cortex. B. Percentage of cells with an abnormal distribution of Cdc15p. The cells were grown in YES or YES supplemented with 1.2 M sorbitol, 1.0 M KCl or 0.2 M MgCl_2_ for 15 minutes and collected by filtration (YES, Sorbitol, KCl, and MgCl_2_, respectively), allowed to grow until they reached the end of the logarithmic phase (3.5×10^8^ cells/ml), and collected by filtration (Late logarithmic), or were collected by centrifugation when they were growing actively in YES medium (Centrifugation). The standard deviation is given for each value. C. Left panel, percentage of cells from the indicated strains showing an abnormal distribution of Cdc15-GFP when cultured in YES or YES with sorbitol at the indicated temperatures. The standard deviation is given for each value. Right panel, micrographs of cells cultured in the presence of sorbitol for 15 minutes at 32°C. Bar, 10 µm.

These results suggested that CAR was unstable and sensitive to stress in the *cfh3Δ* mutant. In order to confirm this hypothesis, we observed cells from the WT or the *cfh3Δ* strains that had been subjected to different stress conditions under the microscope: these conditions were osmotic stress (incubation with 1.2 M sorbitol, 1 M KCl or 0.2 M MgCl_2_ for 15 minutes at 32°C), nutritional stress (growth until late logarithmic phase), and mechanical stress (centrifugation for 2 minutes at 16000× g). In all cases we found cells with an abnormal localization of Cdc15p, which included asymmetric rings (50% of cases; arrow in figure 3 A); rings that did not disassemble properly (20% of cases; figure 3 A, asterisk), broken rings (25% of cases; bracket in figure 3 A) or an accumulation of the protein in the lateral cell cortex (5% of cases; arrowhead in figure 3 A). A closer analysis of CARs with confocal microscopy allowed us to confirm that when the *cfh3Δ* strain was exposed to a stress source, a certain percentage of rings were misshapen, including rings that were asymmetric, broken, and/or distorted (two examples are shown in figure 3 A, lower panels).

When the total number of the cells with an abnormal distribution of Cdc15p was quantified for each strain and condition (n≥500 in all cases), it was found that in the *cfh3Δ* cultures this number was significantly higher than in the WT cultures, even when the cells were growing in logarithmic phase in YES medium, and that this number increased dramatically in the mutant strain when the cells were stressed (figure 3 B). Similar results were obtained using Cdc4 and Rlc1 GFP-fused proteins (abnormal Cdc4 rings were detected in 0.4% of WT cells and in 10% of *cfh3Δ* cells grown in YES, and in 33% of WT cells and in 85% of *cfh3Δ* cells incubated in YES with 1 M KCl for 15 minutes. The values for cells carrying Rlc1-GFP were 0.5%, 1.8%, 52%, and 69%, respectively). These results showed that the contractile rings were less stable in the *cfh3Δ* mutant than in the WT strain, particularly when the cells were undergoing some stress.

### Contractile rings in *cps1-191* cells are sensitive to stress

As described above, we found that the *cfh3^+^* gene played a role in maintaining CAR stability. Since Cfh3p is a regulator of the -glucan synthase Bgs1p [32], we wondered whether both functions of the Cfh3 protein were related. To analyze this, we observed the Cdc15-GFP rings in the WT, *cfh3Δ*, *cps1-191*, and *cfh3Δ cps1-191* strains that had been incubated in YES medium at 25°C (a permissive temperature for the *cps1-191* mutation) or at 32°C (a semi-restrictive temperature for *cps1-191*; this temperature allowed us to observe rings at different stages of contraction and to detect differences between the *cps1-191*, and *cfh3Δ cps1-191* strains, which was not possible at 36°C), or in YES plus 1.2 M sorbitol (osmotic stress) for 15 minutes at 32°C. We quantified the total number of cells exhibiting an abnormal distribution of Cdc15-GFP, as explained above (a minimum of 500 cells were scored in each case). Quantification of the abnormal distribution of Cdc15 in these strains revealed that in all conditions the *cps1-191* mutant exhibited more cells with abnormal rings than the *cfh3Δ* mutant, and that the *cps1-191 cfh3Δ* double mutant showed the strongest defect (figure 3 C). Thus, 38% of the *cps1-191* cells exhibited abnormal Cdc15 rings when they grew under the permissive temperature; this defect was observed in 63% of the cells incubated at 32°C and in up to 82% of the cells when the culture had been subjected to osmotic shock (figure 3 C). The percentages of cells with abnormal rings for the *cfh3Δ cps1-191* strain were 42%, 77%, and 91% for the YES cultures incubated at 25°C or at 32°C, and for the YES plus sorbitol culture incubated at 32°C, respectively. The right panels in figure 3 C show micrographs of the Cdc15 rings in the WT, *cfh3Δ*, *cps1-191* and *cfh3Δ cps1-191* strains grown in YES medium at 32°C. When the cells were incubated in the presence of 1 M KCl instead of sorbitol, similar results were obtained (not shown). Figure S5 shows Cdc4-GFP rings in the WT, *cfh3Δ*, *cps1-191* and *cfh3Δ cps1-191* strains incubated at 25°C. These results showed that a defective Bgs1 protein led to a defect in the stability of the CAR and that this phenotype was enhanced when the cells lacked Cfh3p and when they underwent a stress shock, and suggested that the defects in the CAR observed in the *cfh3Δ* strain might be the consequence of the misregulation of Bgs1p in this mutant.

### Cfh3p ensures Bgs1p stability at the septal area but not at the cell poles

**Figure 4:**
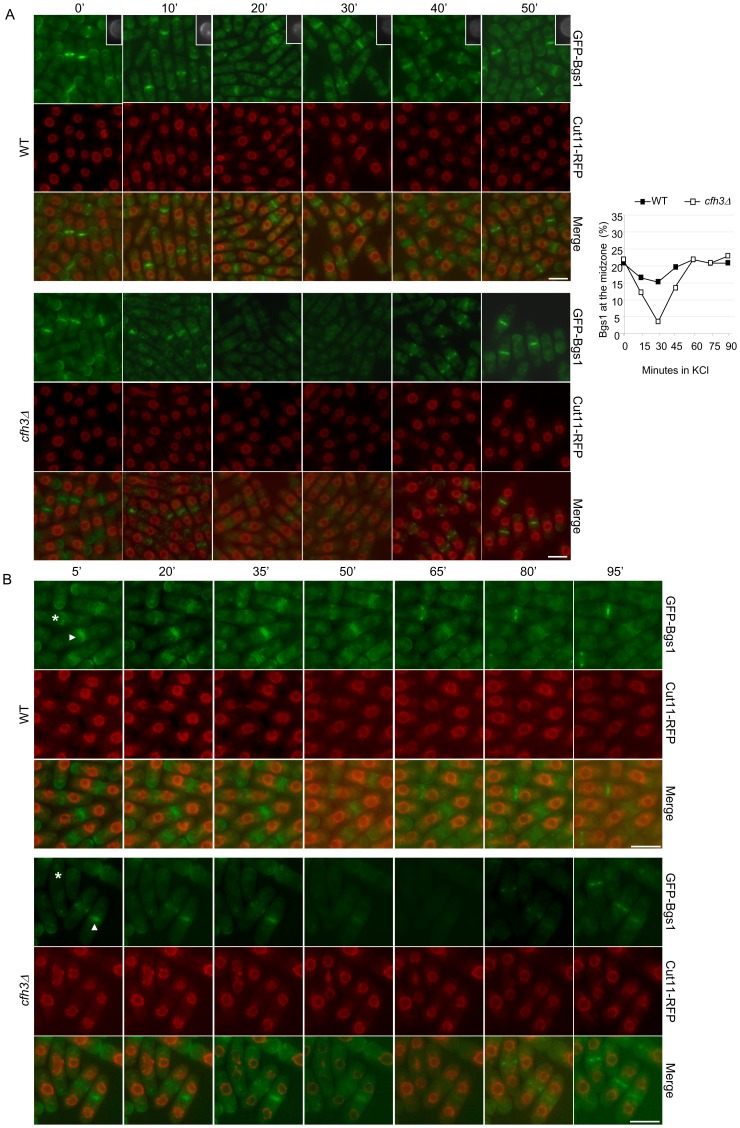
Effect of osmotic shock in the localization of Bgs1p. A. Left panels, wild-type or *cfh3Δ* cells bearing GFP-tagged Bgs1p and RFP-tagged Cut11p were incubated in the presence of 1 M KCl for the indicated times, collected by filtration and photographed. The insets show cell poles. Right panel, cells from the same strains were treated with 1 M KCl; samples were collected by filtration at the indicated times and photographed. The percentage of cells exhibiting GFP-Bgs1 in the cell midzone (with respect to the total cell number) was scored from the photographs. The experiment was performed three times, with similar results; the result of a representative experiment is shown. B. Time-lapse experiment of cells from the same strains treated with 1 M KCl, collected by filtration, spread onto YES+1 M KCl on a slide and photographed along time; the numbers indicate the minutes, after KCl had been added, at which the cells were photographed. Cells in which the Bgs1 ring was starting and finishing assembly/contraction at the 5′ time-point are marked by an asterisk and an arrowhead respectively. Bar, 10 µm.

The physical interaction between Cfh3p and the CAR suggested that Cfh3p could act as a scaffold required to ensure the stability of Bgs1p at the cell equator. In order to investigate this possibility, cells from the WT strain or the *cfh3Δ* mutant bearing Cut11-RFP (a nuclear-membrane protein used as a cell-cycle marker) and GFP-Bgs1 proteins were exposed to 1 M KCl and incubated at 32°C for different times. As shown in figure 4, in the WT strain the GFP-Bgs1 protein could be observed at the cell poles and at the septal area in the control culture (cells incubated in YES medium; marked as 0′ in figure 4 A) and at 10 minutes after KCl had been added to the medium. After 20–30 minutes of incubation in the presence of the salt, the fluorescence corresponding to GFP-Bgs1p was strong at the cell midzone but very weak or undetectable at the poles see insets in the upper panels of figure 4 A). According to the florescence signal, the septal area was distorted when the cells were incubated in the presence of KCl for 10–30 minutes. After longer incubation times (40–50 minutes), Bgs1p was observed at the cell equator and also at the poles; this signal probably corresponded to new Bgs1p molecules that were delivered to the membrane after the initial osmotic shock. The fluorescence observed at the poles after 40–50 minutes of incubation in the presence of KCl was not as strong as that observed in the cells incubated in the absence of KCl (upper panels in figure 4 A and results not shown), perhaps due to an enhanced endocytosis of Bgs1p under stress conditions [32]. Additionally, at this time (40–50 minutes) the septal area was not distorted. In the *cfh3Δ* strain, Bgs1p was observed at the cell equator and the poles when the cells were incubated in YES medium and at 10 minutes after the addition of KCl to the culture (figure 4 A, lower panels). After 20–30 minutes in the presence of KCl, Bgs1 could not be observed either at the cell midzone or at the poles in most cells, in agreement with previous results [32]. After 40 minutes, the GFP signal was seen at the cell midzone and the poles. Thus, Cfh3p is critical for ensuring the presence of Bgs1p in the septal area after stress shock.

In order to quantify these results, the percentage of cells with the GFP-Bgs1 fluorescence signal at the cell midzone with respect to the total cell number was scored at different times after the addition of 1 M KCl to the cultures; the results, shown in the right panel of figure 4 A, confirmed that in the *cfh3Δ* mutant the number of cells exhibiting GFP-Bgs1 in the cell midzone decreased dramatically after osmotic shock, while this treatment had a milder effect in the WT control. Additionally, the results confirmed that GFP-Bgs1p was present in the cell midzone of *cfh3Δ* cells after 45–60 minutes of incubation in the presence of KCl.

Time-lapse experiments were performed to observe the effect of osmotic shock along time in the same cells. Under the conditions of these experiments, no re-localization of GFP-Bgs1p to the cell poles was observed, and all the process seemed to proceed more slowly than in liquid medium. However, the results confirmed that in the WT strain Bgs1p was present in the septal area of the cells along the experiment, while in the *cfh3Δ* mutant the fluorescence signal disappeared from the cell equator after the stress shock and was observed again at later times (figure 4 B).

### Cells restore the cytokinesis machinery after the initial stress shock

**Figure 5:**
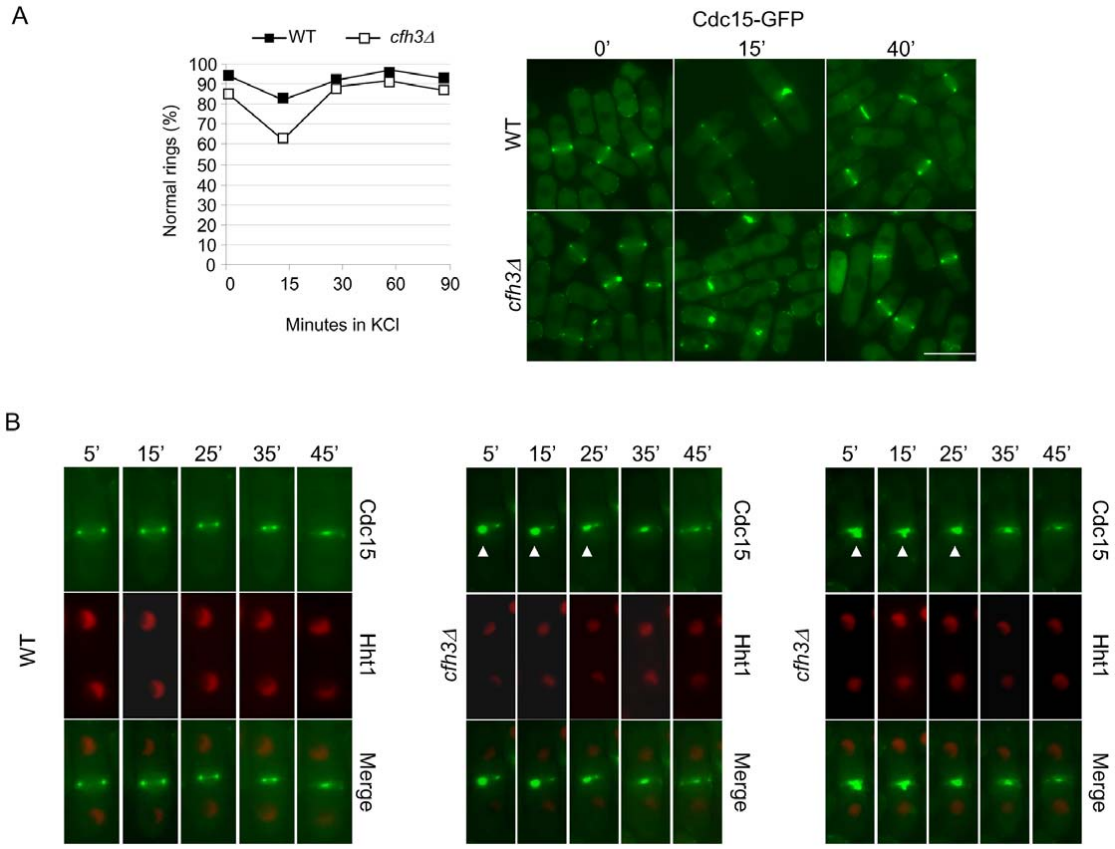
Cells repair the damage produced to the contractile ring by osmotic shock. A. Left panel, Wild-type or *cfh3Δ* cells bearing GFP-tagged Cdc15p were treated with 1 M KCl; samples were collected by filtration at the indicated times and photographed. The percentage of dividing cells with a normal distribution of Cdc15-GFP (with respect to the total number of cells exhibiting Cdc15 in the cell midzone) was scored from the photographs. The experiment was performed three times, with similar results; the result of a representative experiment is shown. Right panel, cells from the same strains were incubated in the presence of 1 M KCl for the indicated times, collected by filtration and photographed. Bar, 10 µm. B. Time-lapse experiments of WT (left set of photographs) and *cfh3Δ* (central and right sets of photographs) cells bearing Cdc15-GFP and Hht1-RFP that were treated with 1 M KCl, collected by filtration, spread onto YES+1 M KCl on a slide and photographed along time; the numbers indicate the minutes, after KCl was added, at which the cells were photographed. Arrowheads point to abnormal rings.

As described above, GFP-Bgs1p was observed in the cell midzone of *cfh3Δ* cells after 40 minutes of incubation in the presence of KCl. Additionally, at this time GFP-Bgs1 localized to the cell poles in both the WT and *cfh3Δ* strains and the septal area was not distorted. In order to determine whether this adaptation to the stress insult was specific to the localization of Bgs1p, we analyzed CAR morphology in WT and *cfh3Δ* cells bearing the Cdc15-GFP fusion protein incubated in the presence of KCl for different times. The number of cells exhibiting normal Cdc15 rings with respect to the number of cells with normal and abnormal rings (such as those shown in figure 3 A) was calculated (cells in interphase were not scored). The plot in the left panel of figure 5 A shows a quantification of the results. The number of cells with normal rings decreased in the WT and *cfh3Δ* strains after 15 minutes of incubation in the presence of the salt; as described above (figure 3), CARs were more affected by osmotic shock in the *cfh3Δ* than in the WT strain. 30 minutes after the osmotic shock, in both strains the number of cells exhibiting a normal CAR was similar to that obtained when the cells were incubated in YES medium (0′ time-point). The right panel in figure 5 A shows WT and *cfh3Δ* cells bearing Cdc15-GFP that had been treated with KCl for different times; similar results were obtained when the cells had been treated with 1.2 M Sorbitol instead of KCl (not shown). These results showed that cells were able to restore the contractile rings after osmotic shock.

In order to follow the recovery of the CAR in a single cell we performed time-lapse experiments in WT and *cfh3Δ* cells bearing both the Cdc15 ring protein fused to the GFP and the Hht1p histone (used as a cell cycle marker) fused to the RFP. Figure 5 B shows the behavior of one WT cell (left set of micrographs) and two *cfh3Δ* cells (central and right sets of micrographs) incubated in YES with 1 M KCl; the *cfh3Δ* cells exhibited asymmetric/broken rings 5 minutes after the addition of the salt (indicated with arrowheads). In both cases, the CARs behaved as normal rings after 25 minutes in the presence of KCl. These results confirmed that cells were able to remedy the damage produced to the cytokinesis apparatus by the initial stress shock and to proceed through cell division.

**Figure 6:**
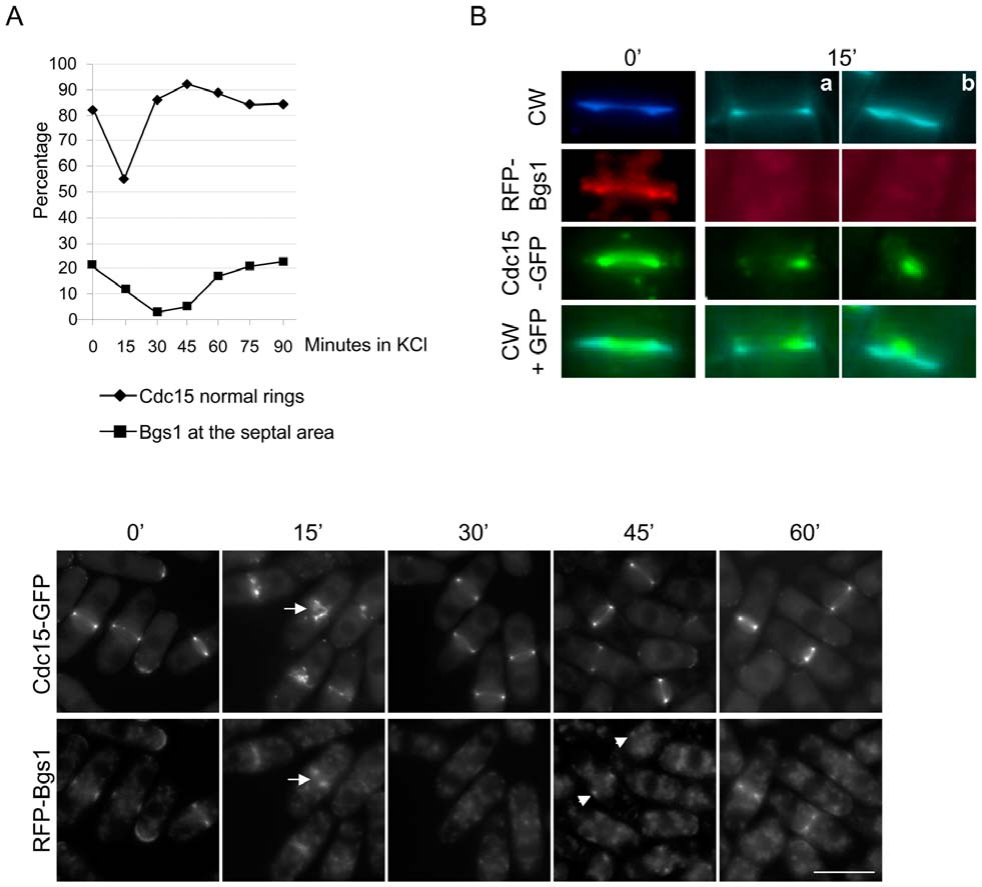
Analysis of the damage produced to the cytokinesis machinery by osmotic shock. A. Upper panel, *cfh3Δ* cells bearing both Cdc15-GFP and RFP-Bgs1 fusion proteins were treated with 1 M KCl; samples were collected by filtration at the indicated times and photographed. The percentage of dividing cells with a normal distribution of Cdc15-GFP (with respect to the total number of cells exhibiting Cdc15 in the cell midzone) and the percentage of cells exhibiting RFP-Bgs1 in the cell midzone (with respect to the total cell number) were scored from the photographs. The experiment was performed three times, with similar results; the result of a representative experiment is shown. Lower panel, micrographs showing *cfh3Δ* cells bearing both the Cdc15-GFP and RFP-Bgs1 proteins that had been grown in YES supplemented with 1 M KCl for the indicated times. The arrows in the panel corresponding to the 15′ time-point mark the septal area of a cell with aberrant Cdc15 and Bgs1 rings. The arrowheads in the panel corresponding to the 45′ time-point mark a weak RFP-Bgs1 signal at the cell equator. Bar, 10 µm. B. Micrographs showing the septal area of *cfh3Δ* cells bearing both the Cdc15-GFP and RFP-Bgs1 proteins grown in YES supplemented with 1 M KCl for the indicated times and stained with Calcofluor White (CW); a and b, septal area of two different cells incubated in the presence of KCl for 15 minutes.

In order to determine whether CAR integrity and the localization of Bgs1p in the septal area were restored simultaneously or consecutively, we cultured a *cfh3Δ* strain bearing both the Cdc15-GFP and the RFP-Bgs1 fusion proteins in YES with 1 M KCl and analyzed both processes in the same culture. As shown in figure 6 A, upper panel, the number of cells with normal Cdc15 rings decreased significantly 15 minutes after the addition of KCl, in agreement with previous results (figures 3 and 5); after 30 minutes in the presence of KCl, the percentage of cells with normal rings was similar to that scored in YES medium (0′ time-point). With respect to Bgs1p, this protein was in the cell midzone in 23% of the cells cultured in YES medium; 15 minutes after addition of KCl, this number decreased to 12%, and it fell to 3% after 30 minutes in the presence of the salt. This percentage increased slightly after 45 minutes and was 18% after 60 minutes. These results showed that stress produced a fast and dramatic damage in CAR integrity and that the cells could repair this damage very efficiently. Regarding Bgs1, both its delocalization in response to stress shock and its re-localization to the cell midzone took place more gradually. The lower panel in figure 6 A shows representative fields of *cfh3Δ* cells bearing the Cdc15-GFP and RFP-Bgs1 proteins that had been incubated in YES with 1 M KCl for different times. The photographs show that 15 minutes after osmotic shock cells exhibited aberrant CARs; Bgs1p was still observed at the cell equator, although the RFP signal did not form a neat ring, as it did when the cells were incubated in YES medium (see the cell marked by an arrow in the lower panel of figure 6 A). Thus, although the morphology of the contractile rings is perturbed by stress, CARs seem to be competent to retain ring-associated proteins at the cell equator. After 30 minutes in the presence of the salt, CARs were normal and cells did not exhibit Bgs1p in the cell midzone. The RFP-Bgs1 ring was observed in some cells after 45 minutes (indicated with arrowheads in the figure 6 A, lower panel) and it was present in all the dividing cells after 60 minutes in the presence of KCl. It was not possible to perform time-lapse experiments to analyze CAR recovery and Bgs1p re-localization in the same cell because the RFP-Bgs1 fluorescence was very weak in the presence of KCl and faded before the end of the experiment.

The *cfh3Δ* strain bearing the Cdc15-GFP and RFP-Bgs1 proteins allowed us to analyze the effect of stress on cytokinesis in more detail. In YES medium (0′), most mitotic cells exhibited Cdc15p as a contractile ring located at the leading edge of the growing septum (CW in figure 6 B). The Cdc15 ring coincided with the Bgs1 signal, which was observed as a contractile ring at the leading edge of the growing septum; Bgs1 left a fluorescent signal behind as it contracted. Under stress conditions, there were cells with Cdc15 rings that did not display the Bgs1 signal, and cells in which the Cdc15 signal was not located at the leading edge of the growing septa (two examples are shown in Figure 6 B). These results confirmed that stress collapsed and discoordinated the cytokinesis machinery.

## Discussion

### Cfh3p and cytokinesis

We have previously described that Cfh3p regulates the activity of the (1,3)glucan synthase Bgs1p, particularly under stress conditions [32]. In this work we aimed to further characterize the function of this protein. The time at which Cfh3p localized to the division site, and the fact that it formed a contractile ring pointed to a role of Cfh3p in cytokinesis. *cfh3^+^* overexpression led to defects in cell division; analysis of this phenotype did not provide information about this role, since the phenotype was accompanied by an aberrant distribution of many proteins required for different steps of cytokinesis. The physical interaction between Cfh3p and Cdc15p, a ring-associated protein, suggested that the defects in the cytokinesis machinery observed in cells overexpressing *cfh3^+^* might be indirect; an excess of Cfh3p probably disturbs the structural/mechanical properties of a structure that is dynamic and highly regulated. Even so, a specific role for Cfh3p in CAR assembly/contraction can be inferred from the facts that *cfh3Δ* mutants showed a genetic interaction with mutants defective in ring assembly/contraction and that in a *cfh3Δ* mutant a significant number of cells exhibited abnormal contractile rings. Cfh3p interacts physically with Bgs1p [32] and with the CAR (figure 2); thus, Cfh3p might act as a scaffold whose interaction with Cdc15p and/or other CAR component or CAR-associated proteins would be required for Bgs1p to become stabilized at the plasma membrane at the site of cell division.

### Cfh3p, Bgs1p, and cytokinesis under stress

In a *cps1-191* mutant, a significant number of cells had abnormal CARs, even at the permissive temperature, which suggested that the (1,3)glucan synthase *bgs1^+^*/*cps1^+^* is required for CAR stability. Most interestingly, we found that in the WT strain CARs were unstable under osmotic, nutritional and mechanical stress conditions, and that the effect of stress was more dramatic in the *cfh3Δ*, the *cps1-191* and the *cfh3Δ cps1-191* cells. In the absence of Cfh3p, the activity of Bgs1p is reduced due to an enhanced endocytosis, particularly after stress shock [32]. This strongly suggested that the damage to the CAR observed in the *cfh3Δ* strains could be explained in terms of the defect in Bgs1p of this mutant. Thus, in the WT strain a fully functional Bgs1p would be delivered to the membrane and would remain there for the time required to exert its activity at a normal rate. In the *cfh3Δ* mutant, a robust Bgs1p would be delivered to the membrane and would act properly for some time, but this protein would be endocytosed faster than in the WT strain. This would result in a lower functionality of the (1,3)glucan synthase and in the appearance of subtle CAR defects. In the *cps1-191* strain, a weak Bgs1 protein would be delivered such that although it could remain at the membrane for a normal length of time, it would lead to some cell defects. Finally, in the *cfh3Δ cps1-191* double mutant, a defective Bgs1 protein would be delivered to the membrane and endocytosed faster than in the single *cps1-191* mutant, resulting in very low Bgs1p functionality, which would account for the strong defects detected in this strain ([32] and this work). The defects in these strains would be exacerbated by stress, which reduces the stability of Bgs1p at the plasma membrane. We observed that in the WT strain Bgs1p delocalized from the cell poles but not from the cell equator after stress shock, and that in the *cfh3Δ* mutant Bgs1p delocalized from both, cell poles and midzone. Thus, Cfh3p is essential to guarantee that linear -glucan is synthesized correctly at the primary septum (where it plays its most relevant function; [27]), even under unfavorable conditions.

### Contractile ring, primary septum, and cytokinesis under stress

It seems plausible to think that defective CARs present in the cells after stress shock and/or in the *cps1-191* mutant could be a consequence of defects in the synthesis of the primary septa. In *Saccharomyces cerevisiae*, coordination between the synthesis of a chitin primary septum and the contraction of the acto-myosin ring is required to overcome the internal turgor pressure during cell division and for the cell to proceed successfully through cytokinesis [38]. ScChs2p, the chitin synthase required for primary septum synthesis [39], is also required to maintain CAR stability [40]. In *S. pombe*, the (1,3)glucan synthase Bgs1p is required for the correct synthesis of the primary septum [27], which is made up of glucan. The fact that the *bgs1*/*cps1-191* mutant had abnormal CARs (even when grown in YES medium) could be explained if the weak activity in this mutant synthesized defective primary septa unable to support contraction, thus reducing the stability of the CAR. This defect would be enhanced by stress due to the reduced stability of Bgs1p at the plasma membrane, in particular in the *cfh3Δ* mutant. However, the observations that even in a WT strain the number of defective rings increased after a short osmotic shock, that in the *cfh3Δ* mutant this phenomenon was observed at a time at which Bgs1p was still observed in the cell midzone (15 minutes), and that the rings were restored before Bgs1p re-localized to the septal area suggest that stress might induce direct damage to the contractile ring. Consequently, in the *cfh3Δ* cells a combination of two effects produced by stress (direct damage to the CAR and a defective septum synthesis due to the reduced Bgs1p activity) would result in a defect in CAR stability stronger than that produced in the WT strain, which would only be affected by the direct damage produced to the ring by stress, a defect that is rapidly repaired by the cell. In the case of the *cps1-191* mutants, the cells would have a weak primary septum, even in YES medium, which would result in the presence of some defective rings; under these circumstances, the direct damage produced to the CAR by a stress shock would have strong consequences, and would account for the severe defects in the cytokinesis apparatus detected in the in the *cps1-191* and *cfh3Δ cps1-191* strains after osmotic shock.

### The contractile ring as a sensor for stress

Our results show that stress collapses the cytokinesis machinery. Previous results had shown that stress produces alterations in other morphogenetic elements in different organisms; thus, actin becomes depolarized and the dynamics of microtubules is affected by osmotic shock [41]–[45]. It has been proposed that the reorganization of actin after osmotic shock would be a protective response directed to reinforcing the cell cortex after the cell shrinkage produced by the change in external osmolarity [43]. It is likely that the depolarization of actin after centrifugation [46] would also be a protective response to the mechanical stress produced during that process. When cells are under hyper-osmotic conditions they shrink and the membrane undergoes changes in its physical state and in protein-protein and protein-lipid interactions [47], [48]. It is possible that these circumstances might affect the cytokinesis machinery. After a certain time of incubation in the hyper-osmotic condition, cells become adapted to the new environment by adjusting their internal osmolarity; they reorganize the distribution of actin and restore microtubule dynamics and tip growth [41], [45], [49]. We found that cells were able to recover from the initial osmotic shock; after a prolonged incubation under stress, cells stabilized the contractile rings and re-localized Bgs1p to the cell division site and cell poles. It is possible that the rapid damage produced to the CAR could trigger a mechanism that would promote cell adaptation to osmotic stress and repair of the cytokinesis machinery. Once the CAR has been restored, Bgs1p would be relocated to the septal area and septum synthesis would reinitiate. These results are in agreement with the fact that neither the WT nor the *cfh3Δ* strains exhibit defects in cytokinesis under these conditions ([32] and this work). Thus, the contractile ring could be considered as a sensor that detects environmental conditions and promotes protective responses to ensure the accuracy of cell division.

It has been described that Cdc15p dephosphorylation is required for its functionality at the CAR [50]. We analyzed whether osmotic shock promoted Cdc15p phosphorylation and CAR recovery was concomitant with Cdc15p dephosphorylation in the WT and *cfh3Δ* strains; we found that Cdc15p mobility was not slower in extracts obtained from cells incubated with KCl than in extracts obtained from the control culture (not shown). This suggested that there was no correlation between CAR instability after osmotic shock and Cdc15p phosphorylation, and seemed to rule out the possibility that the adaptation mechanism involved changes in Cdc15p phosphorylation. Thus, although changes in Cdc15 phosphorylation cannot be completely excluded, it is possible that other processes could guarantee the stability of the contractile rings under stress conditions. Determining their nature should shed light on the mechanisms that guarantee cell division in unfavorable environments.

## Materials and Methods

### General techniques

All techniques for *S. pombe* growth and manipulation have been described ([51], http://www.biotwiki.org/foswiki/bin/view/Pombe/NurseLabManual). The source and relevant genotypes of the strains used are listed in Table S1. Unless stated, cells were incubated at 32°C. To induce osmotic stress, either powdered KCl was added to the culture at the desired final concentration or the cells were collected by filtration and transferred to YES supplemented with 1.2 M sorbitol. For overexpression experiments using the *nmt1^+^* promoter in the pREP3X plasmid, cells were grown in EMM medium containing appropriate supplements and 15 µM thiamine; cells were harvested, washed extensively with water, and resuspended in EMM with supplements. For phenotype analysis, expression was induced for 20–24 hours. In order to express *cfh3^+^* from the thiamine-repressible *nmt1^+^* promoter, site-directed mutagenesis was used to introduce an *Xho*I site immediately upstream from the initial ATG. The *cfh3^+^* ORF and 1 kb of the 3′non-coding sequence were then cloned into the overexpression pREP3X plasmid as an *Xho*I/*Sac*I DNA fragment. Geneticin (G418, ForMedium) and hygromycin (ForMedium) were used at 120 and 400 µg ml^−1^, respectively. Molecular and genetic manipulations were according to Sambrook *et al.* [52]. All tagged proteins were integrated into the chromosome under the control of their own promoters. Double mutants were obtained by tetrad analysis. Combinations of mutated alleles with HA-, GFP- or RFP-tagged proteins were performed either by plasmid transformation or by “random spore” selection from genetic crosses [51].

### Protein techniques

Western blotting and co-immunoprecipitations were performed as described [32].

### Microscopy

Hoechst binds preferentially to the A/T-rich zones in DNA. We used Hoechst 33258 because its slow entry into the cells allows a non-specific staining of the cell wall. Thus, simultaneous observation of the nuclei and the cell wall can be performed in living cells. Unless stated, the observation of tagged proteins was performed on cells collected by filtration. In order to estimate the percentage of cells with damaged cytokinesis machinery, samples were collected at the desired times and photographed. The percentage of cells exhibiting normal Cdc15-GFP ring morphology and GFP-Bgs1 localization in the cell midzone was scored from the photographs. In the former case, only cells exhibiting contractile rings were scored, while in the latter all cells in the field were scored. The experiments were performed a minimum of three times and a minimum of 500 cells were scored in each experiment. For conventional fluorescence microscopy, images were captured with a Leica DM RXA microscope equipped with a Photometrics Sensys CCD camera, using the Qfish 2.3 program. Confocal microscopy was performed with a Leica TCS SL spectral confocal microscope with a 63×1.4 oil objective, using an excitation wavelength of 488 nm. Images were processed with Adobe Photoshop or Leica Confocal Software.

## Supporting Information

Figure S1 **Localization of proteins involved in different stages of cytokinesis in cells overexpressing** ***cfh3^+^*** **.** For comparison, the distribution of the different proteins in the WT strain is shown in the left-hand side panels of each set of pictures. (A) Cell wall staining with Calcofluor (left panels) and actin staining with rhodamine-phalloidin (right panels). (B–F) For each set of micrographs the panel on the left shows nuclear and cell wall staining with Hoechst 33258, and the panel on the right shows the GFP fluorescence signal. (B) Distribution of the myosin light-chain Cdc4p. The asterisk marks a cell in which the Cdc4 protein can be observed in the midzone after the septum has been synthesized; the dot marks a cell in which a new ring has been assembled close to a previous ring that has not contracted completely, and the arrow points to an abnormal distribution of Cdc4p at the cell cortex. (C) Distribution of the PCH protein Cdc15p. The arrows point to an abnormal distribution of Cdc15p at the cell cortex; the dot marks a cell in which a second ring has been assembled in the body of a cell that has not undergone cell separation, and the asterisk marks an asymmetric ring. (D) Distribution of the chitin synthase-like Chs2p. The arrows point to the position where there should be a Chs2p ring and the asterisk marks an asymmetric ring. (E) Localization of the Spn3p septin. The arrows point to abnormal accumulation of the protein at the cell cortex and cytoplasm. The enlarged set of panels on the right shows Hoechst staining (left panel), the GFP signal (central panel), and the merged image (right panel) of the midzone area of a cell with multiple septa, in which the growing septa push the septin ring into the cell. (F) Distribution of Agn1p glucanase. The dots mark different positions at which the GFP signal should be observed. Bar, 10 µm.
(TIF)

Figure S2 **Phenotype of cells overexpressing** ***cfh3^+^*** **and** ***bgs1^+^*** **.** A. Distribution of GFP-Cfh3p and RFP-Bgs1p in cells expressing *cfh3^+^* from its endogenous promoter (upper panels) or from the 3X*nmt1^+^* promoter (lower panels). B. Hoechst 33258 staining of cells overexpressing *cfh3^+^* and *bgs1^+^*. Bar, 10 µm.
(TIF)

Figure S3 **Phenotype of cells overexpressing** ***cfh3^+^*** **and** ***chs2^+^*** **.** A. Cells from the indicated strains bearing the pREP3X*cfh3^+^* plasmid were incubated in the presence (promoter OFF) or absence (promoter ON) of thiamine for 10 hours; the cultures were then transferred to 36°C and incubated for an additional 12 hours. Arrows point to cells with multiple septa. B. Cells bearing the indicated plasmids were incubated in the absence of thiamine for 22 hours at 32°C. A and B, cells were stained with Hoechst and photographed. Bar, 10 µm.
(TIF)

Figure S4 **Time of Cfh3p arrival at the cell equato**r. The photographs show time-lapse experiments of strains bearing Sad1-GFP (a spindle-pole body protein), Cdc15-GFP, and RFP-Bgs1 (A); Cdc15-GFP and RFP-Cfh3 (B), or GFP-Cfh3 and RFP-Bgs1 (C). Arrows point to the first appearance of the corresponding protein. Numbers indicate the minutes at which the pictures were taken. Bar, 10 µm.
(TIF)

Figure S5 **Localization of Cdc4p in the indicated strains incubated in YES medium at 25°C.** Arrows point to abnormal rings. Bar, 10 µm.
(TIF)

Table S1 **Yeast strains used in this study.**
(PDF)
