# Correction: The Integrity of the Cytokinesis Machinery under Stress Conditions Requires the Glucan Synthase Bgs1p and Its Regulator Cfh3p

**DOI:** 10.1371/annotation/67c14ddf-301d-4f96-be92-eb2586fd8ac8

**Published:** 2013-09-23

**Authors:** Mohammad Reza Sharifmoghadam, M.-Ángeles Curto, Marta Hoya, Nagore de León, Rebeca Martin-Garcia, Cristina Doncel, M.-Henar Valdivieso

There were errors in the Funding section. The correct funding information is as follows: Financial support to the Instituto de Biología Funcional y Genómica (IBFG) from the Fundación Ramón Areces and to our group from the Comisión Interministerial de Ciencia y Tecnología (CICYT) (Spain)/European Union FEDER (Fondo Europeo de Desarrollo Regional) program (grant BFU2007-61866 and BFU2010-15085) made this work possible. Dr. Curto was supported by a contract for training in research and technological innovation by Junta de Castilla y León and by Formacion de Personal Investigador (FPU) fellowship. Dr. de Leon and Dr. Hoya were supported by FPU and Junta de Ampliacion de Estudios (JAE)-PREDOC fellowships from the Ministry of Science and Consejo Superior de Investigaciones Científicas (CSIC), Spain, respectively. The funders had no role in study design, data collection and analysis, decision to publish, or preparation of the manuscript. 

