# Supplementary figures and images for: The Integrity of the Cytokinesis Machinery under Stress Conditions Requires the Glucan Synthase Bgs1p and Its Regulator Cfh3p
Source: PLoS One. 2012 Aug 15;7(8):e42726. doi: 10.1371/journal.pone.0042726 (PMC3419747; doi:10.1371/journal.pone.0042726)

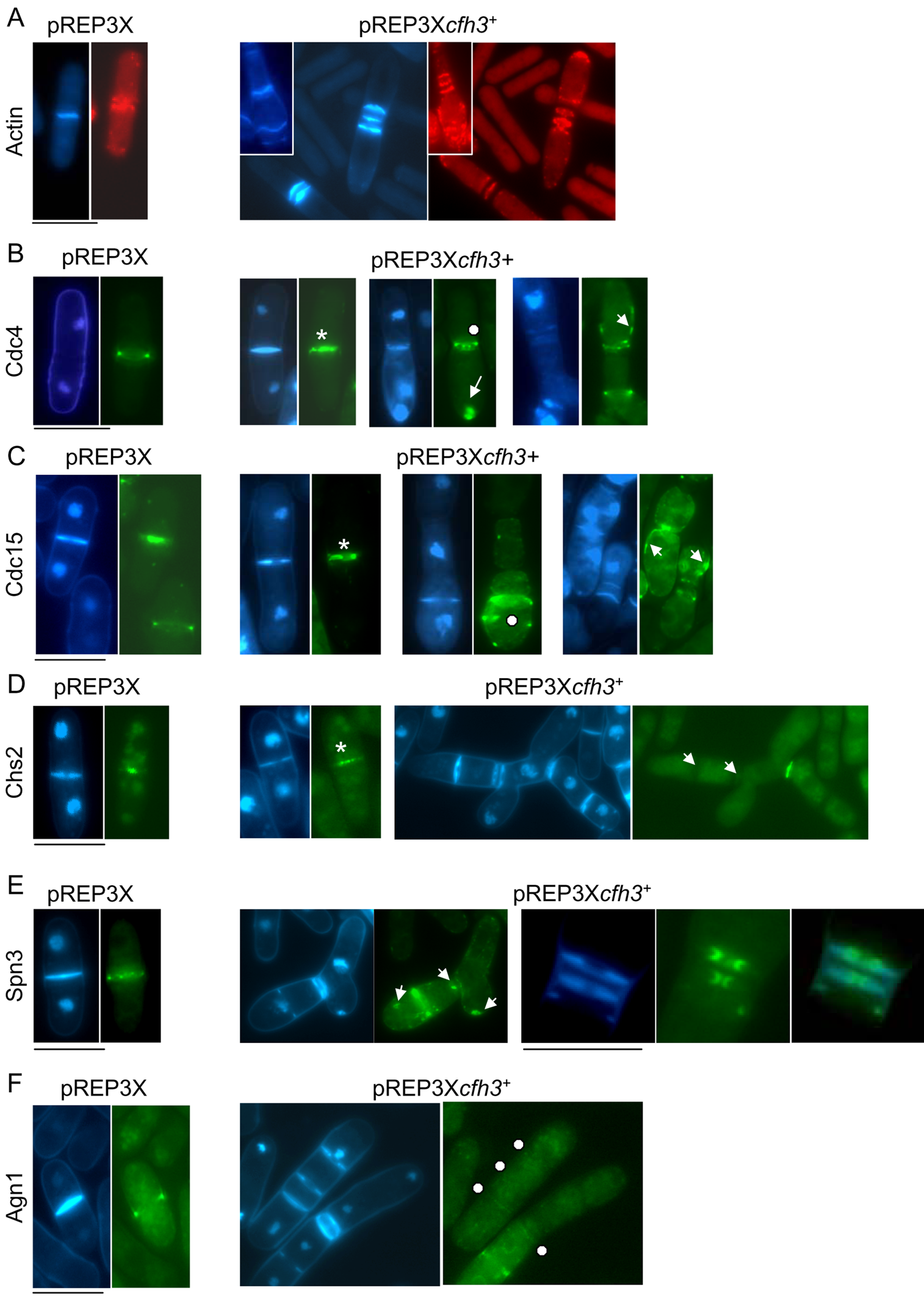

Supplement: Figure S1 — Localization of proteins involved in different stages of cytokinesis in cells overexpressing cfh3+ . For comparison, the distribution of the different proteins in the WT strain is shown in the left-hand side panels of each set of pictures. (A) Cell wall staining with Calcofluor (left panels) and actin staining with rhodamine-phalloidin (right panels). (B–F) For each set of micrographs the panel on the left shows nuclear and cell wall staining with Hoechst 33258, and the panel on the right shows the GFP fluorescence signal. (B) Distribution of the myosin light-chain Cdc4p. The asterisk marks a cell in which the Cdc4 protein can be observed in the midzone after the septum has been synthesized; the dot marks a cell in which a new ring has been assembled close to a previous ring that has not contracted completely, and the arrow points to an abnormal distribution of Cdc4p at the cell cortex. (C) Distribution of the PCH protein Cdc15p. The arrows point to an abnormal distribution of Cdc15p at the cell cortex; the dot marks a cell in which a second ring has been assembled in the body of a cell that has not undergone cell separation, and the asterisk marks an asymmetric ring. (D) Distribution of the chitin synthase-like Chs2p. The arrows point to the position where there should be a Chs2p ring and the asterisk marks an asymmetric ring. (E) Localization of the Spn3p septin. The arrows point to abnormal accumulation of the protein at the cell cortex and cytoplasm. The enlarged set of panels on the right shows Hoechst staining (left panel), the GFP signal (central panel), and the merged image (right panel) of the midzone area of a cell with multiple septa, in which the growing septa push the septin ring into the cell. (F) Distribution of Agn1p glucanase. The dots mark different positions at which the GFP signal should be observed. Bar, 10 µm. (TIF) [file pone.0042726.s001.tif]

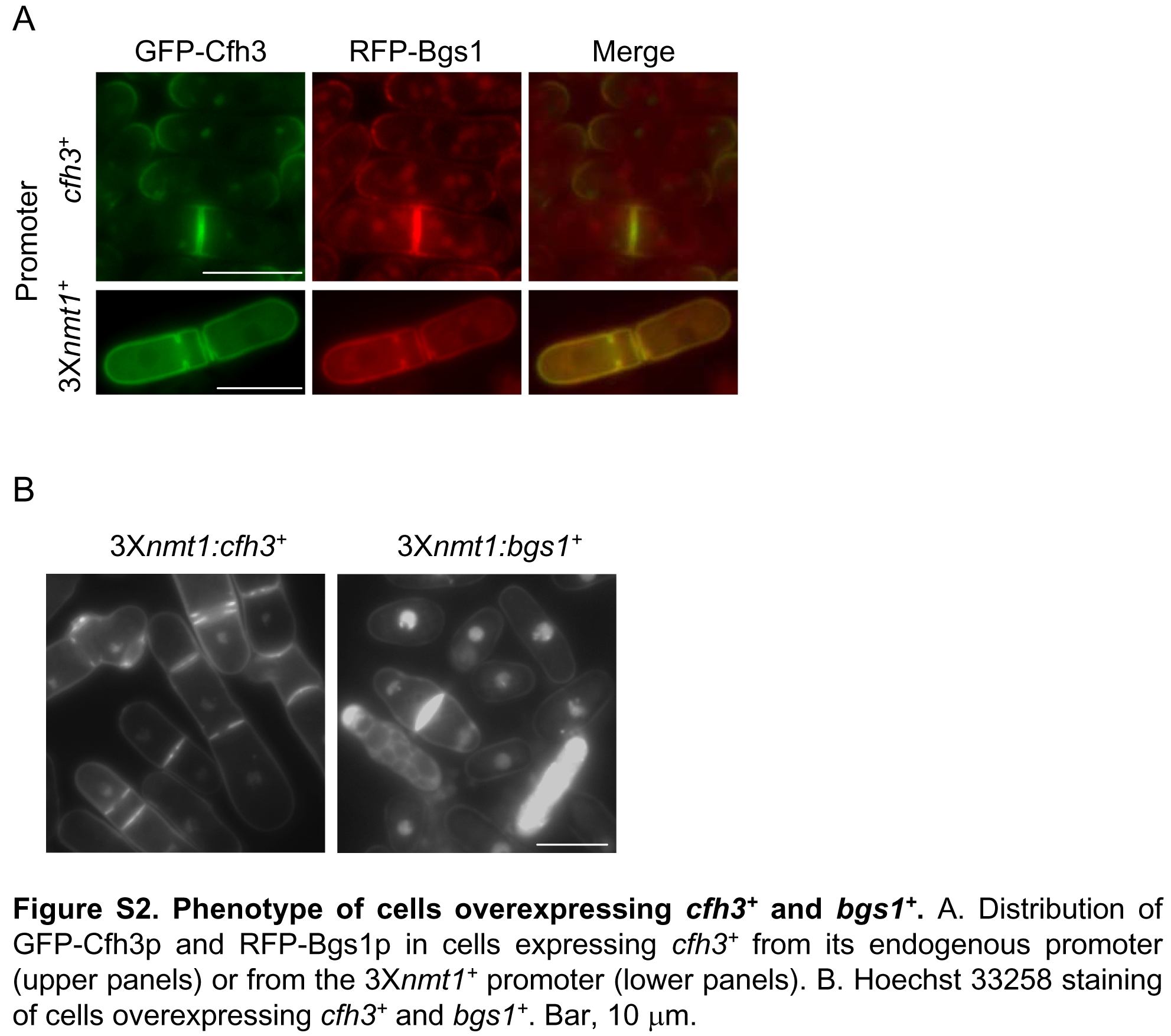

Supplement: Figure S2 — Phenotype of cells overexpressing cfh3+ and bgs1+ . A. Distribution of GFP-Cfh3p and RFP-Bgs1p in cells expressing cfh3+ from its endogenous promoter (upper panels) or from the 3Xnmt1+ promoter (lower panels). B. Hoechst 33258 staining of cells overexpressing cfh3+ and bgs1+. Bar, 10 µm. (TIF) [file pone.0042726.s002.tif]

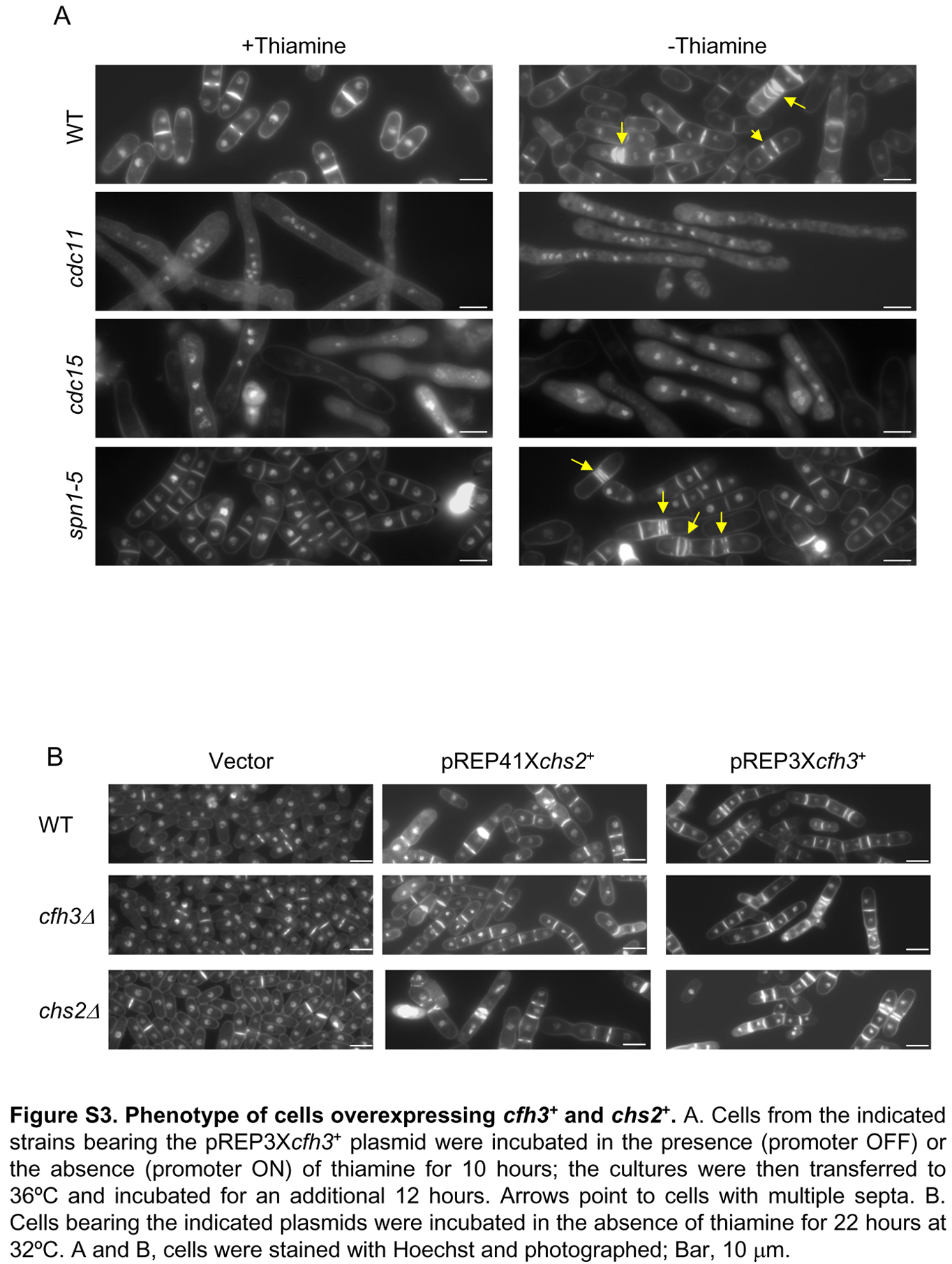

Supplement: Figure S3 — Phenotype of cells overexpressing cfh3+ and chs2+ . A. Cells from the indicated strains bearing the pREP3Xcfh3+ plasmid were incubated in the presence (promoter OFF) or absence (promoter ON) of thiamine for 10 hours; the cultures were then transferred to 36°C and incubated for an additional 12 hours. Arrows point to cells with multiple septa. B. Cells bearing the indicated plasmids were incubated in the absence of thiamine for 22 hours at 32°C. A and B, cells were stained with Hoechst and photographed. Bar, 10 µm. (TIF) [file pone.0042726.s003.tif]

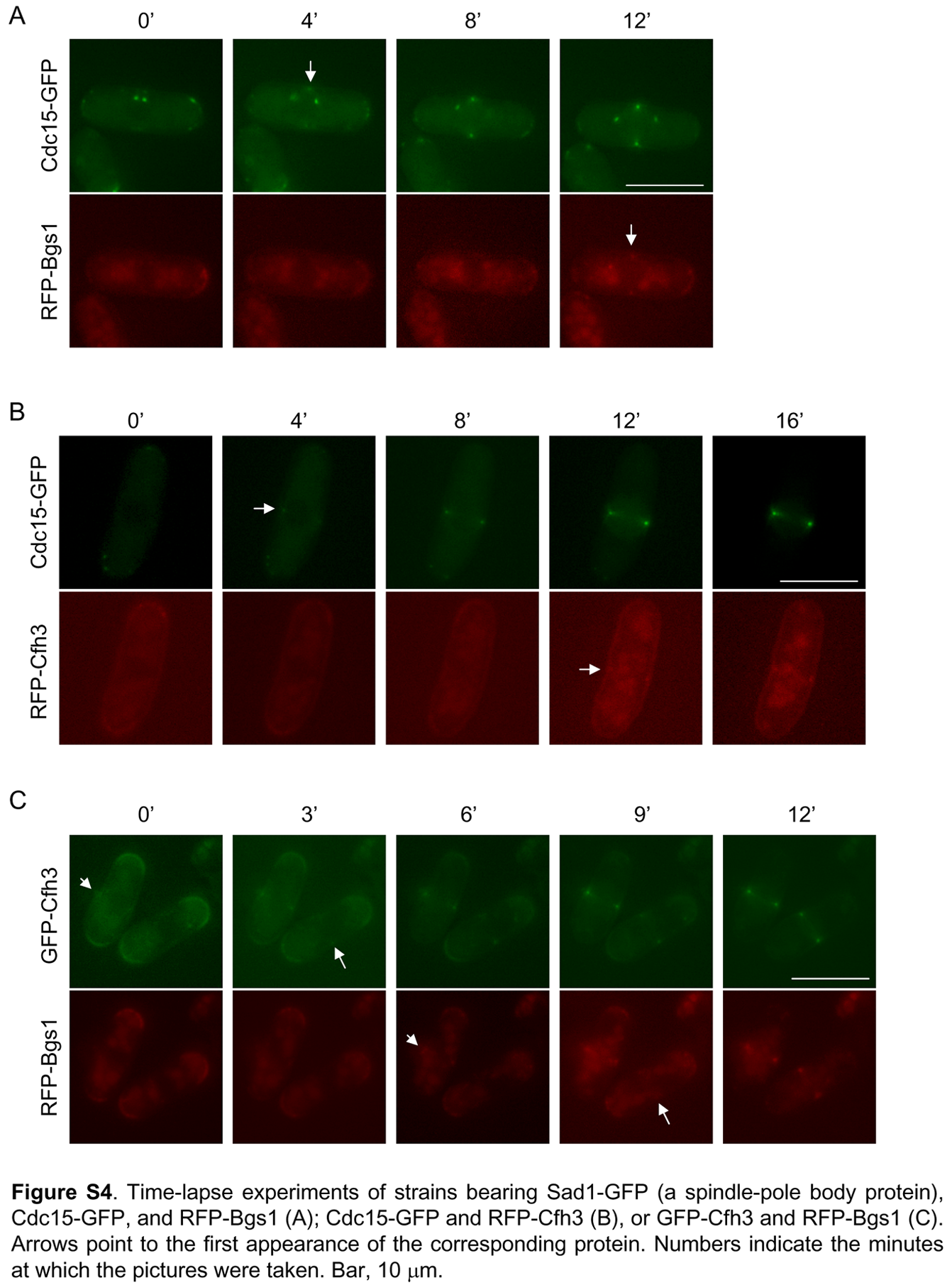

Supplement: Figure S4 — Time of Cfh3p arrival at the cell equator. The photographs show time-lapse experiments of strains bearing Sad1-GFP (a spindle-pole body protein), Cdc15-GFP, and RFP-Bgs1 (A); Cdc15-GFP and RFP-Cfh3 (B), or GFP-Cfh3 and RFP-Bgs1 (C). Arrows point to the first appearance of the corresponding protein. Numbers indicate the minutes at which the pictures were taken. Bar, 10 µm. (TIF) [file pone.0042726.s004.tif]

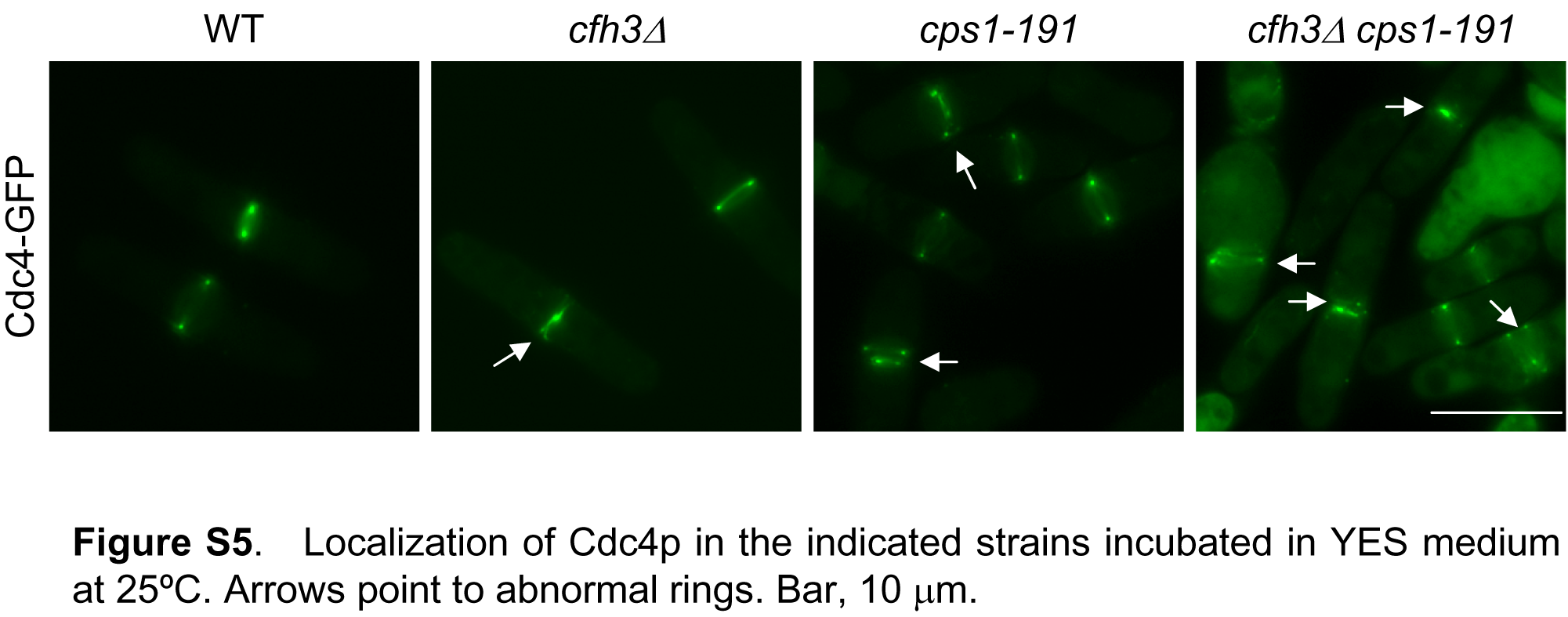

Supplement: Figure S5 — Localization of Cdc4p in the indicated strains incubated in YES medium at 25°C. Arrows point to abnormal rings. Bar, 10 µm. (TIF) [file pone.0042726.s005.tif]
