# Supplementary material for: The Integrity of the Cytokinesis Machinery under Stress Conditions Requires the Glucan Synthase Bgs1p and Its Regulator Cfh3p
Source: PLoS One. 2012 Aug 15;7(8):e42726. doi: 10.1371/journal.pone.0042726 (PMC3419747; doi:10.1371/journal.pone.0042726)
Supplement: Table S1 — Yeast strains used in this study. (PDF) [file pone.0042726.s006.pdf]

**Table S1: Strains used in this work**

| STRAIN | GENOTYPE | SOURCE |
|---|---|---|
| HVP30 | leu1-32 his3-Δ1 ura4Δ18 ade6 h$^{-}$ | Lab. stock |
| HVP75 | leu1-32 h$^{-}$ | Lab stock |
| HVP162 | leu1-32 his3-Δ1 ade6 h$^{-}$ | Lab. stock |
| HVP280 | chs2::ura4$^{+}$ leu1-32 ura4-Δ18 his3-Δ1 ade6 h$^{-}$ | Lab. stock |
| HVP458 | leu1-32 h$^{-}$ | Lab stock |
| HVP495 | leu1-32 h$^{-}$ pREP3Xcfh3$^{+}$ | Lab stock |
| HVP513 | spn3::ura4$^{+}$ leu1-32 ade6 h$^{+}$ | Jurg Bähler |
| HVP514 | spn4::ura4$^{+}$ leu1-32 ade6 h$^{-}$ | Jurg Bähler |
| HVP515 | spn1-5:ura4+ ade6 h$^{-}$ | Jurg Bähler |
| HVP530 | cdc11-119 leu1-32 h- | P. Nurse |
| HVP531 | cdc14-118 leu1-32 h- | P. Nurse |
| HVP532 | cdc15-140 leu1-32 h$^{-}$ | P. Nurse |
| HVP533 | cdc16-116 leu1-32 h$^{+}$ | P. Nurse |
| HVP543 | chs2::ura4$^{+}$ :chs2-GFP:leu1$^{+}$ ura4-Δ18 his3-Δ1 ade6 h$^{-}$ | Lab. stock |
| HVP607 | imp2::ura4$^{+}$ leu1-32 ade6-M216 | Shelly Sazer |
| HVP630 | cfh3::KAN leu1-32 his3Δ1 ura4-Δ18 ade6 h$^{-}$ | Lab stock |
| HVP631 | cfh3::KAN leu1-32 his3Δ1 ura4-Δ18 ade6 h$^{-}$ | Lab stock |
| HVP642 | myo3::ADE2 leu1-32 ura4D18 h$^{+}$ | I. Mabuchi |
| HVP652 | cdc4-8 leu1-32 h$^{+}$ | P. Nurse |
| HVP707 | myo2-E1leu1-32 ura4D18 his3D1 ade6 h$^{-}$ | M. Balasubramanian |
| HVP783 | chs2::ura4$^{+}$ leu1-32 ura4-Δ18 his3-Δ1 ade6 h$^{-}$ pREP3Xcfh3$^{+}$ | This work |
| HVP784 | cfh3::KAN leu1-32 his3Δ1 ura4-Δ18 ade6 h$^{-}$ pREP3Xchs2$^{+}$ | This work |
| HVP786 | myo2E1 myo3::ADE2 leu1-32 ura4-Δ18 h$^{+}$ | Lab stock |
| HVP819 | cps8-188 leu1-32 h$^{+}$ | P. Perez |
| HVP994 | cdc15-GFP:ura4$^{+}$ leu1-32 ura4-Δ18 h$^{-}$ | S. Moreno |
| HVP999 | eng1-GFP:KAN leu1-32 ura4-Δ18 ade6 h$^{-}$ | CR Vazquez |
| HVP1000 | agn1-GFP:KAN leu1-32 ura4-Δ18 ade6 h$^{-}$ | CRVazquez |
| HVP1051 | cdc4-GFP::ura4$^{+}$ leu1-32 ade6 h$^{-}$ | M. Balasubramanian |
| HVP1053 | rlc1-GFP:KAN leu1-32 ura4-Δ18 h$^{-}$ | P. Perez |
| HVP1054 | cdc4-GFP::ura4$^{+}$ leu1-32 ade6 h$^{-}$ pREP3X | This work |
| HVP1098 | hht2-GFP:ura4$^{+}$ leu1-32 ade6 h$^{+}$ | M. Balasubramanian |
| HVP1153 | cps1-191 leu1-32 ura4Δ18 h$^{-}$ | M. Balasubramanian |
| HVP1213 | spn3-GFP:KAN leu1-32 ura4-Δ18 ade6 h$^{+}$ | M. Balasubramanian |
| HVP1230 | spn3-GFP:KAN leu1-32 ura4-Δ18 ade6 h$^{+}$ pREP3Xcfh3$^{+}$ | This work |
| HVP1231 | cdc4-GFP::ura4$^{+}$ leu1-32 ade6 h$^{-}$ pREP3Xcfh3$^{+}$ | This work |
| HVP1232 | cdc15-GFP:ura4$^{+}$ leu1-32 ura4-Δ18 h$^{-}$ pREP3Xcfh3$^{+}$ | This work |
| HVP1233 | agn1-GFP:KAN leu1-32 ura4-Δ18 ade6 h$^{-}$ pREP3Xcfh3$^{+}$ | This work |
| HVP1244 | rlc1::KAN leu1-32 ura4-Δ18 ade6 h$^{-}$ | V. Simanis |
| HVP1247 | chs2::ura4$^{+}$ leu1-32 ura4D18 his3D1 ade6M210 h$^{-}$ pALchs2-GFP pJRH3X | This work |
| HVP1248 | chs2::ura4$^{+}$ leu1-32 ura4D18 his3D1 ade6M210 h$^{-}$ pALchs2-GFP pJRH3Xcfh3$^{+}$ | This work |
| HVP1255 | agn1-GFP:KAN leu1-32 ura4-Δ18 ade6 h$^{-}$ pREP3X | This work |
| HVP1256 | cdc15-GFP:ura4$^{+}$ leu1-32 ura4-Δ18 h$^{-}$ pREP3X | This work |
| HVP1257 | spn3-GFP:KAN leu1-32 ura4-Δ18 ade6 h$^{+}$ pREP3X | This work |

| | | |
|---|---|---|
| HVP1281 | *cfh3::KAN GFP-cfh3:leu1*$^{+}$ *leu1-32 ura4D18his3D1 ade6M210 h*$^{-}$ | Lab stock |
| HVP1316 | *cdc16-116 GFP-cfh3:leu1*$^{+}$*h*$^{+}$ | This work |
| HVP1330 | *myo2-E1 myo3::ADE2 GFP-cfh3:leu1*$^{+}$ *leu1-32 ura4D18h*$^{+}$ | This work |
| HVP1358 | *cdc4-GFP:ura4*$^{+}$ *cfh3::KAN leu1-32ura4D18 ade6 h*$^{+}$ | This work |
| HVP1359 | *cdc15-GFP:ura4*$^{+}$ *cfh3::KAN leu1-32 ura4D18 h*$^{+}$ | This work |
| HVP1369 | *cdc11-119 leu1-32 h*$^{-}$ pREP3X*cfh3*$^{+}$ | This work |
| HVP1402 | *spn1-5:ura4+ ade6 h*$^{-}$ pREP3X*cfh3*$^{+}$KANMX6 | This work |
| HVP1464 | *cdc15-140 leu1-32 h*$^{-}$ pREP3X*cfh3*$^{+}$ | This work |
| HVP1471 | *bgs1::ura4*$^{+}$ *Pbgs1:GFP-bgs1:leu1*$^{+}$ *his3-Δ1 h*$^{-}$ | J.C. Ribas |
| HVP1563 | *HA-cfh3:leu1*$^{+}$ *leu1-32 h*$^{-}$ | Lab stock |
| HVP1581 | *cdc15-140 cfh3::KAN leu1-32 h*$^{+}$ | This work |
| HVP1603 | *imp2::ura4 cfh3::KAN leu1-32 ade6-M216 h*$^{+}$ | This work |
| HVP1610 | *cps8-188 cfh3::KAN leu1-32 h*$^{+}$ | This work |
| HVP1652 | *cps1-191 cdc4-GFP:ura4*$^{+}$ *leu1-32 ura4D18 ade6* | This work |
| HVP1655 | *cps1-191 leu1-32 ura4Δ18 h*$^{-}$ pREP3X*cfh3*$^{+}$ | This work |
| HVP1686 | *cps1-191 cfh3::KAN cdc4-GFP:ura4*$^{+}$ *leu1-32 ura4D18 ade6* | This work |
| HVP1706 | *cps1-191 GFP-cdc15 leu1-32 ura4D18 h* | This work |
| HVP1709 | *cps1-191 cfh3::KAN GFP-cdc15 leu1-32 ura4D18 h* | This work |
| HVP1718 | *bgs1::ura4 GFP-Bgs1:leu1*$^{+}$ *RFP-Bgs1KAN leu1-32 ura4D18 his3D1 h*$^{+}$ | This work |
| HVP1775 | *cdc15-GFP:ura4*$^{+}$ *HA-cfh3 :leu1*$^{+}$ *leu1-32 ura4-Δ18 h*$^{-}$ | This work |
| HVP1810 | *cdc15-GFP:ura4*$^{+}$ *RFP-bgs1 :leu1*$^{+}$ *leu1-32 ura4-Δ18 h*$^{-}$ | This work |
| HVP1811 | *cdc15-GFP :ura4*$^{+}$ *RFP-Bgs1:leu1*$^{+}$ *cfh3 ::KAN leu1-32 ura4D18 h*$^{+}$ | This work |
| HVP2031 | *sad1-GFP :KAN leu1-32 ura4D18 ade6 h*$^{+}$ | P. Perez |
| HVP2177 | s*ty1::ura4*$^{+}$ *leu1-32 ade6-M216 h*$^{+}$ | Y. Sanchez |
| HVP2367 | *GFP-atb2:KAN cut11-RFP:Hygr sfi1-CFP:Natr leu1-32 ura4D18 his2 h*$^{+}$ | Yeast Genetic Resource Center (Japan) |
| HVP2434 | *Hht1-mRFP:KAN h*$^{-}$ | J. Cooper |
| HVP2714 | *leu1-32 h*$^{-}$ *pREP3Xbgs1*$^{+}$ | This work |
| HVP2904 | *RFP-Bgs1:leu1*$^{+}$*GFP-cfh3:ura4*$^{+}$ *leu1-32 ura4D18 his3D1 ade6M210 h*$^{-}$ | This work |
| HVP2987 | *sad1-GFP:KAN cdc15-GFP :ura4*$^{+}$ *RFP-bgs1 :leu1*$^{+}$ *leu1-32 ura4D18 ade6* | This work |
| HVP2898 | *cfh3::KAN bgs1::ura4*$^{+}$ *GFP-bgs1:leu cut11-RFP: Hygr leu1-32 ura4D18 his-* | This work |
| HVP3004 | *cdc11-119 GFP-cfh3:leu atb2-GFP:KAN cut11-RFP: Hygr* | This work |
| HVP3005 | *cdc15-140 GFP-cfh3:leu atb2-GFP:KAN cut11-RFP: Hygr* | This work |
| HVP3023 | *bgs1::ura4*$^{+}$ *GFP-bgs1:leu cut11-RFP: Hygr leu1-32 ura4D18 his-* | This work |
| HVP3099 | *cut11-RFP:Hygr GFP-Cfh3:leu1*$^{+}$ | This work |
| HVP3121 | *hht1-mRFP:KAN cdc15-GFP:ura* | This work |
| HVP3122 | *hht1-mRFP:KAN cdc15-GFP:ura cfh3::KAN* | This work |
